# Towards quantifying the glacial runoff signal in the freshwater input to Tyrolerfjord–Young Sound, NE Greenland

**DOI:** 10.1007/s13280-016-0876-4

**Published:** 2017-01-23

**Authors:** Michele Citterio, Mikael K. Sejr, Peter L. Langen, Ruth H. Mottram, Jakob Abermann, Signe Hillerup Larsen, Kirstine Skov, Magnus Lund

**Affiliations:** 1 Glaciology and Climate Department, Geological Survey of Denmark and Greenland (GEUS), Øster Voldgade 10, 1350 Copenhagen K, Denmark; 2 Arctic Research Centre, Aarhus University, Ny Munkegade bldg. 1540, 8000 Aarhus C, Denmark; 3 Danish Meteorological Institute (DMI), Lyngbyvej 100, 2100 Copenhagen Ø, Denmark; 4 Asiaq Greenland Survey, Qatserisut 8, 3900 Nuuk, Greenland; 5 Department of Geosciences and Natural Resource Management, University of Copenhagen, Øster Voldgade 10, 1350 Copenhagen K, Denmark; 6 Department of Bioscience, Arctic Research Centre, Aarhus University, Frederiksborgvej 399, 4000 Roskilde, Denmark

**Keywords:** Glacial runoff, Greenland, Modelling, Observations, Surface salinity

## Abstract

Terrestrial freshwater runoff strongly influences physical and biogeochemical processes at the fjord scale and can have global impacts when considered at the Greenland scale. We investigate the performance of the HIRHAM5 regional climate model over the catchments delivering freshwater to Tyrolerfjord and Young Sound by comparing to the unique Greenland Ecological Monitoring database of in situ observations from this region. Based on these findings, we estimate and discuss the fraction of runoff originating from glacierized and non-glacierized land delivered at the daily scale between 1996 and 2008. We find that glaciers contributed on average 50–80% of annual terrestrial runoff when considering different sections of Tyrolerfjord–Young Sound, but snowpack depletion on land and consequently runoff happens about one month earlier in the model than observed in the field. The temporal shift in the model is a likely explanation why summer surface salinity in the inner fjord did not correlate to modelled runoff.

**Electronic supplementary material:**

The online version of this article (doi:10.1007/s13280-016-0876-4) contains supplementary material, which is available to authorized users.

## Introduction

Under ongoing and projected climate change, the glacial meltwater contribution to freshwater runoff into fjords will increase and locally enhance nutrient input and biological productivity (Meire et al. 2015) as well as CO_2_ uptake (Sejr et al. 2011; Rysgaard et al. 2012). At the Greenland scale, the recent increase in freshwater fluxes into the North Atlantic (Bamber et al. 2012) can have an impact on ocean circulation (Fichefet et al. 2003) and is increasingly dominated by meltwater runoff from the ice sheet and peripheral glaciers rather than solid ice discharge (van den Broeke et al. 2009; Bolch et al. 2013; Enderlin et al. 2014). Recent advances have been made in assessing glacier meltwater discharge into the sea at the catchment and Greenland scales by combining in situ observations in fjords and along the coast with river discharge series and with remotely sensed proxies such as near surface sediment plumes (McGrath et al. 2010; Chu et al. 2012; Hudson et al. 2014). With knowledge of river discharge, energy balance models can explain in detail the climate drivers of surface meltwater production (van As et al. 2012). However, less understood hydrological processes including refreezing, internal storage and routing through dynamic reservoirs modify the magnitude and timing of meltwater delivery at the glacier margin (Rennermalm et al. 2013), transiently buffering some of the impact on sea level rise (Harper et al. 2012).

**Fig. 1:**
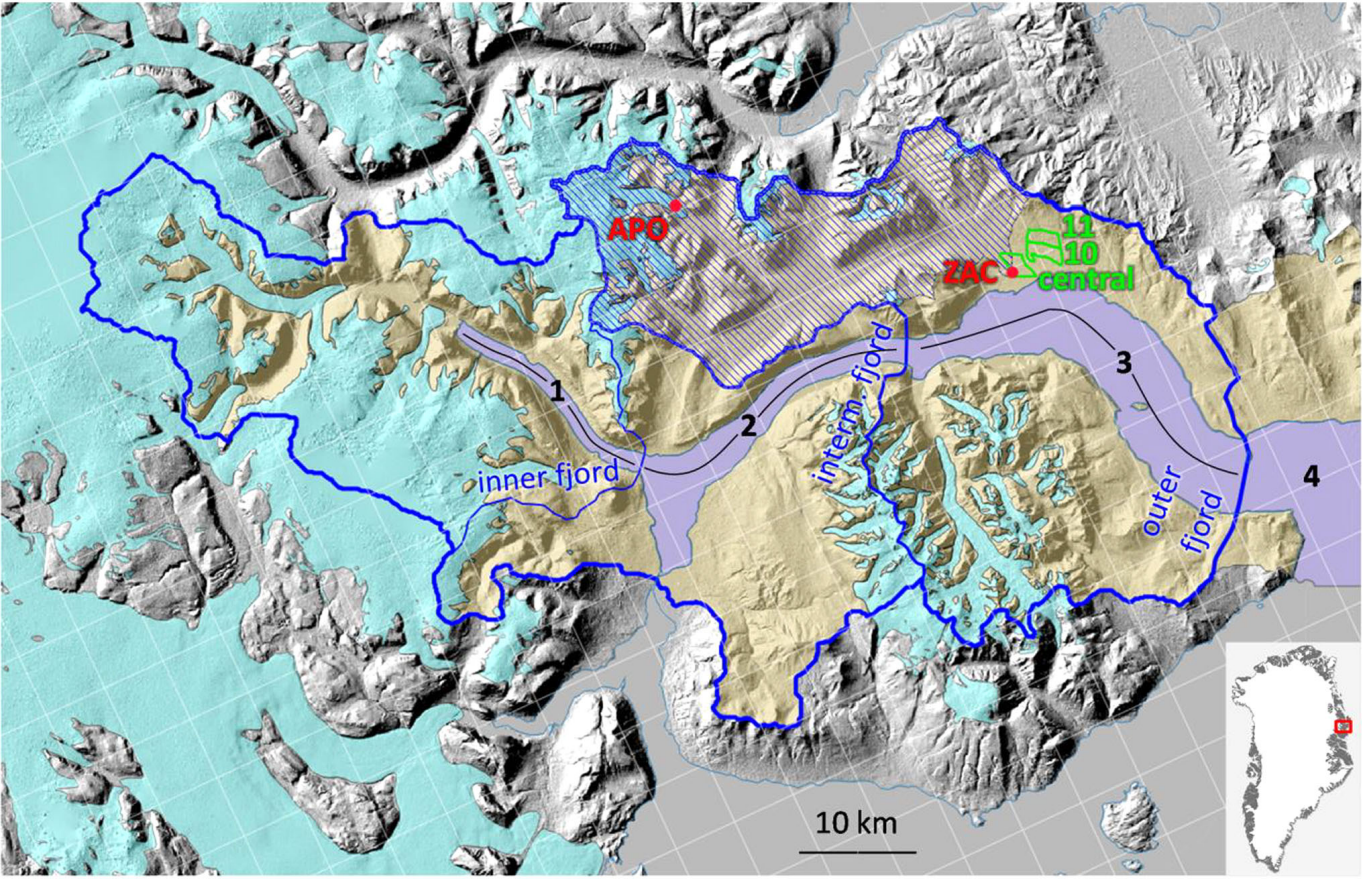
Overview of the study area showing a hillshade visualization of the GIMP DEM. *Light blue, yellow* and *cyan colours* indicate sea, land and glacier surfaces, respectively. The boundary of the hydrological catchments delivering freshwater to the fjord is marked by *thin*, *intermediate* and *thick blue lines*. Diagonal *blue lines* mark the catchment subtended by the Zackenberg River hydrometric station. The *black numbers* and *lines* indicate the fjord segments where salinity measurements are discussed. The *red dots* mark the position of the Zackenberg Station climate mast (‘ZAC’) and of the AWS on the outlet glacier of A.P. Olsen ice cap (‘APO’). Ablation stakes 1 to 9 are installed on this glacier tongue. The *green* polygons and labels indicate the areas used for comparing modelled and observed snow cover depletion curves. The exact footprint of the 0.05° by 0.05° HIRHAM5 grid cells before downscaling is marked by white squares. The map *inset* shows the location of this region in Greenland

Most Greenland fjords receive significant inputs of glacial melt water during summer and this causes an estuarine circulation where low-saline surface water flows out the fjord and is compensated by an inflow of saltier water at depth (Bendtsen et al. 2014). At a local scale, in Greenland coastal waters melt water from the ice sheet has been shown to be an important factor determining the key physical and biological dynamics including the physical mixing of water masses (Mortensen et al. 2011), light and nutrient conditions (Meire et al. 2015; Murray et al. 2015) and primary production (Juul-Pedersen et al. 2015). Finally, the presence of melt water impacts species composition in both the water column and on the sea floor (Sejr et al. 2009; Krawczyk et al. 2015; Arendt et al. 2016). Glacial melt water itself also contains nutrients and bioavailable carbon thus stimulating biological activity (Lawson et al. 2014; Meire et al. 2016). Recent numerical fjord modelling suggests that glacier meltwater runoff into Young Sound and Tyrolerfjord may be 50% higher than previously estimated (Bendtsen et al. 2014). Glacierized catchments in regions characterized by steep topography like Tyrolerfjord (Fig. 1) are challenging to model because large fractions of the lower glacier tongues, where ablation rates are most negative, may be narrower than the grid cell size of even state of the art Regional Climate Models (RCMs). Most studies at the margin of the ice sheet additionally face large uncertainties in defining the glacierized catchment area contributing to runoff (van de Wal and Russell 1994). Surface meltwater typically enters the englacial and subglacial drainage systems quickly (Bartholomew et al. 2011), and poor knowledge of basal topography usually force catchment delineation to rely on surface topography alone. If the basal water pressure is close to the ice overburden pressure, the hydraulic potentiometric surface is indeed predominantly controlled by surface topography (Paterson 1994). However, basal water pressure varies seasonally in response to surface meltwater availability and field observations of basal water pressures are very scarce (Murray and Clarke 1995; Gordon et al. 1998; Sugiyama et al. 2011). By contrast, the Tyrolerfjord–Young Sound hydrological catchment is clearly delineated (Larsen et al. 2012), removing this uncertainty source.

In this paper, we first investigate how temperature, precipitation, snow cover, glacier surface mass balance and surface runoff modelled between 1996 and 2012 by the HIRHAM5 RCM compare to the available in situ observational time series produced by Greenland Ecological Monitoring (GEM), including fjord surface salinity and Zackenberg River discharge. By downscaling the HIRHAM5 grids to a finer spatial resolution, we accurately represent the complex glacier margins and estimate the fraction of freshwater discharge to the sea accounted for by glacier runoff.

## Materials and methods

Seawater observations are available covering the entire length of Tyrolerfjord and Young Sound, but by far most terrestrial monitoring data relevant to this work are concentrated within the Zackenberg River catchment, primarily in the surroundings of Zackenberg Research Station (74.47°N, 20.57°W) and on A.P. Olsen Ice Cap. A campaign to measure freshwater discharge from three different rivers was carried out in 2012 (Larsen et al. 2012), and the surface mass balance of Freya Glacier on Clavering Island has been monitored since 2008 (Hynek et al. 2014), but covering the entire region over several years is only feasible through modelling. The in situ observations used are freely available online at http://data.g-e-m.dk/.

### Terrestrial observations

Meteorological observations are available from automatic weather stations (AWS) at several locations in the Zackenberg River catchment. We use 1996–2012 air temperature at 2 m and precipitation data from the longest meteorological time series, produced by the GEM ClimateBasis Programme at the climate masts close to the Zackenberg Research Station, and 2008–2012 air temperature at 2 m from the lowest AWS operated by the GEM GlacioBasis Programme on the outlet glacier of A.P. Olsen Ice Cap (Fig. 1). For precipitation, which is notoriously difficult to observe accurately, only the years with fewer data gaps have been used. The technical details of these AWS are given, respectively, in Meltofte and Thing (1996) and Citterio et al. (2015). All meteorological observations were temporally resampled to daily average 2 m air temperature and daily precipitation total.

Glacier surface mass balance in the ablation area of the monitored outlet glacier of A.P. Olsen Ice Cap is measured using ablation stakes drilled into the ice and measured down to the ice surface every year in late April. They are re-measured the following year to quantify the amount of ice lost in the intervening ablation season. The surface mass balance observations used in the present study refer to 9 stakes located from 550 to 880 m above sea level (a.s.l.) and all years below the glacier equilibrium line; therefore, no accumulation observations were used. Details of the glacier mass balance programme at A.P. Olsen Ice Cap are given by Citterio et al. (2009).

The spatial distribution of snow cover in the Zackenberg valley has been monitored continuously since 1997 by the GEM GeoBasis Programme using photos obtained from digital cameras mounted at 400 m a.s.l. on an east-facing slope. Details on camera type, settings and pixel resolution can be found in Westergaard-Nielsen et al. (2017). Daily photos, captured each day at solar noon (13:20 UTC), were transformed into digital orthophotos according to Buus-Hinkler et al. (2006), and snow classification and snow depletion curves for pre-defined areas were performed. In this study, we use percent snow cover area observations from the ‘area 10’, ‘area 11’ and ‘central area’ regions covering 3.74, 3.12 and 3.59 km^2^, respectively (Fig. 1).

Zackenberg River discharge is measured close to Zackenberg Research Station and the river mouth. Automatic water level observations are converted using a stage–discharge curve that is established almost every year to account for changes in the riverbed, particularly after the periodic outburst floods originating from A.P. Olsen Land. The catchment upstream of the hydrometric station covers 493 km^2^ of which 92 km^2^ are covered by glaciers. For this study, the discharge observations have been resampled to daily and annual discharge totals. Further details on the discharge measurements are given in Mylius et al. (2014).

### Marine observations

To assess the key physical parameters in the fjord, including the distribution and amount of freshwater, vertical profiles of temperature and salinity were measured each summer at 30–40 (depending on the sea ice conditions) stations in the fjord and on the coastal shelf (Fig. 1) by the GEM MarineBasis Programme. Measurements are conducted in early August using a SeaBird SBE19+ CTD. The instrument is factory-calibrated each year prior to the field campaign and measures at 4 Hz resulting in a vertical resolution of about 25 cm. Data on salinity were averaged for 1-m intervals. Below this surface layer, the salinity varies much less. Spatial variation along the measured transect is influenced by the fjord bathymetry. In the deep basin (section 2), water with a high salinity and density is trapped near the bottom and cannot enter the inner basin (section 1) due to the shallow sill. Similarly, the shallow sill at the entrance of the fjord prevents warm (+1.5 °C) and saline water of Atlantic origin from entering the fjord. To allow comparison of fjord salinity to the modelled runoff, we calculated the average salinity each year from 2004 to 2015. The average salinity was calculated for each of the four sections in the upper 10 metres of the water column.

### HIRHAM5 model

The high-resolution HIRHAM5 RCM is based on the physical parameterizations of the ECHAM5 global climate model (Roeckner et al. 2003) combined with the dynamical scheme of the HIRLAM7 numerical weather prediction model (Eerola 2006). It is run on a rotated polar grid of 0.05° by 0.05° with 31 vertical levels in the atmosphere. The basic model setup is similar to that described in Lucas-Picher et al. (2012) and Rae et al. (2012) with a similar subsurface scheme over glaciers and ice sheets to that described in Langen et al. (2015). There are 25 layers in the subsurface over glaciers to a depth of 70 m water equivalent and includes both ice and snow layers. Snow pack parameterizations account for densification processes as well as retention and refreezing of meltwater within the layers, including the development of perched ice layers. Runoff of liquid meltwater from the snow pack occurs when the pore space is insufficient to hold more liquid water and the cold content of the layer no longer permits refreezing. A “slush-bucket” parameterization gives a slope and time-dependent rate of runoff (Langen et al. accepted).

Comparisons with observations around Greenland show that the model tends to overestimate precipitation at the coast, with a consequent dry bias in the interior of the ice sheet (Lucas-Picher et al. 2012), though this is regionally varying. HIRHAM5 does, however, reproduce temperature observations well, both at the land-based DMI coastal weather stations and at the site on the ice sheet operated by both GC-Net and PROMICE (Rae et al. 2012). Comparison to the PROMICE compilation of historical and current SMB measurements (Machguth et al. 2016) shows that SMB is reliably represented in the model, although very low-elevation, high-ablation rates are underestimated (Langen et al., unpublished).

### Surface elevation, glacier margins and hydrologic catchment boundaries

Topographic information needed in this study is obtained at the 0.05° by 0.05° spatial resolution from the HIRHAM5 DEM (digital elevation model), land/glacier mask and land/ocean mask (Fig. 1). Higher spatial resolution grids of terrain elevation were produced by resampling to the 110 by 110 m cell size used in this study of the 30 by 30 m GIMP (Greenland Mapping Project) version 2.2 DEM, tiles 4.3 and 5.3 (Howat et al. 2014). A constant geoid separation of 32 m was applied over the entire region to approximate terrain elevations above mean sea level. Glacier, land and ocean masks at the 110 by 110 m resolution were rasterized from the PROMICE aerophotogrammetric glacier map of Greenland (Citterio and Ahlstrøm 2013) and from the GEUS vector maps of NE Greenland. The catchments are named in this text and figures as ‘inner’, draining to section 1 of Tyrolerfjord, ‘intermediate’, including the former and draining to include sections 1 and 2 of the fjord, and ‘outer’, including the former two catchments and draining to sections 1, 2, and 3 of Tyrolerfjord and Young Sound. The fourth investigated catchment is that of the Zackenberg River closing at the hydrometric station.

### Downscaling scheme and comparison of model and observations

The HIRHAM5 daily grids of 2-m air temperature, precipitation, snow melt, ice melt, snow water equivalent (SWE), glacier mass balance, glacier runoff and land runoff were nearest neighbour resampled from 0.05° by 0.05° (ca. 5.5 by 5.5 km) to ca. 110 by 110 m. The cell size was chosen to be a factor of 5–10 smaller than the width of the A.P. Olsen Ice Cap outlet glacier tongue where surface mass balance observations are collected. Since Freya Glacier is narrower, its mass balance observations were not used. The HIRHAM5 glacier/land and land/ocean masks were instead replaced with the 110 by 110 m surface type masks gridded from the PROMICE and GEUS vector maps. Missing or excess glacier cover in the 0.05° by 0.05° HIRHAM5 glacier mask was corrected by zeroing the glacier runoff at non-glacier cells or by filling in a glacier runoff estimate from HIRHAM5 glacier cells at similar elevation in the surrounding region (Fig. S2).

Each day, melting versus non-melting HIRHAM5 grid cells were identified based on positive values of either ice or snow melt. Elevation bias results from the vertical separation between the elevation of each 110 by 110 m grid cell and that of the corresponding 0.05° by 0.05° HIRHAM5 cell. For instance, the A.P. Olsen AWS used to compare 2 m air temperature is located at ca. 660 m a.s.l. but HIRHAM5 models the corresponding 0.05° by 0.05° grid cell as laying at 1030 m a.s.l. because of the high topography surrounding the glacier tongue. The elevation bias correction for 2 m air temperature (Fig. S1) was calculated as the elevation difference times the vertical temperature lapse rate estimated daily by least squares fitting of HIRHAM5 elevations and 2 m air temperatures in the surrounding region. These daily temperature lapse rates were estimated separately over melting versus non-melting cells, because a fixed 0 °C surface has a buffering effect on 2 m air temperatures and resulting in different lapse rates. This downscaling approach corrects elevation bias at the sub-RCM cell scale but it does not introduce any regional bias (mean and median of correction over the entire region: −0.09 C and −0.06 C, respectively) and it preserves the HIRHAM5 spatial variability at the scale of the RCM grid cells (Fig. S1).

Extracting model glacier mass balance values comparable with observation is challenging. The HIRHAM5 grid cell where A.P. Olsen ablation stakes 1 to 4 are located is classified as land, not glacier. The cell containing stakes 5 to 9 is modelled at an elevation of 1212 m a.s.l., much higher than the 749–888 m a.s.l. range of these stakes and in most years even higher than the model glacier equilibrium line. We therefore compare instead with the regional annual mass balance averaged over nearby HIRHAM5 grid cells at an elevation within ±50 m from the actual elevation of each measured stake.

Elevation bias correction was not applied to precipitation and SWE because we only compare them to observations close to Zackenberg Research Station where the terrain is less steep and more faithfully represented in the HIRHAM5 DEM.

Daily runoff volumes from the investigated catchments are estimated as the sum over the catchment of land and glacier runoff depths times the true area of each cell, which varies slightly across the region of interest due to the use of a polar stereographic map projection. This effectively assumes that runoff exits the catchment on the same day it exits the HIRHAM5 grid cell it originates from. Runoff was not corrected for elevation bias because doing so would require knowledge of snow-covered versus bare glacier or land surface conditions at the sub-HIRHAM5 cell resolution, which is not available.

## Results

**Fig. 2:**
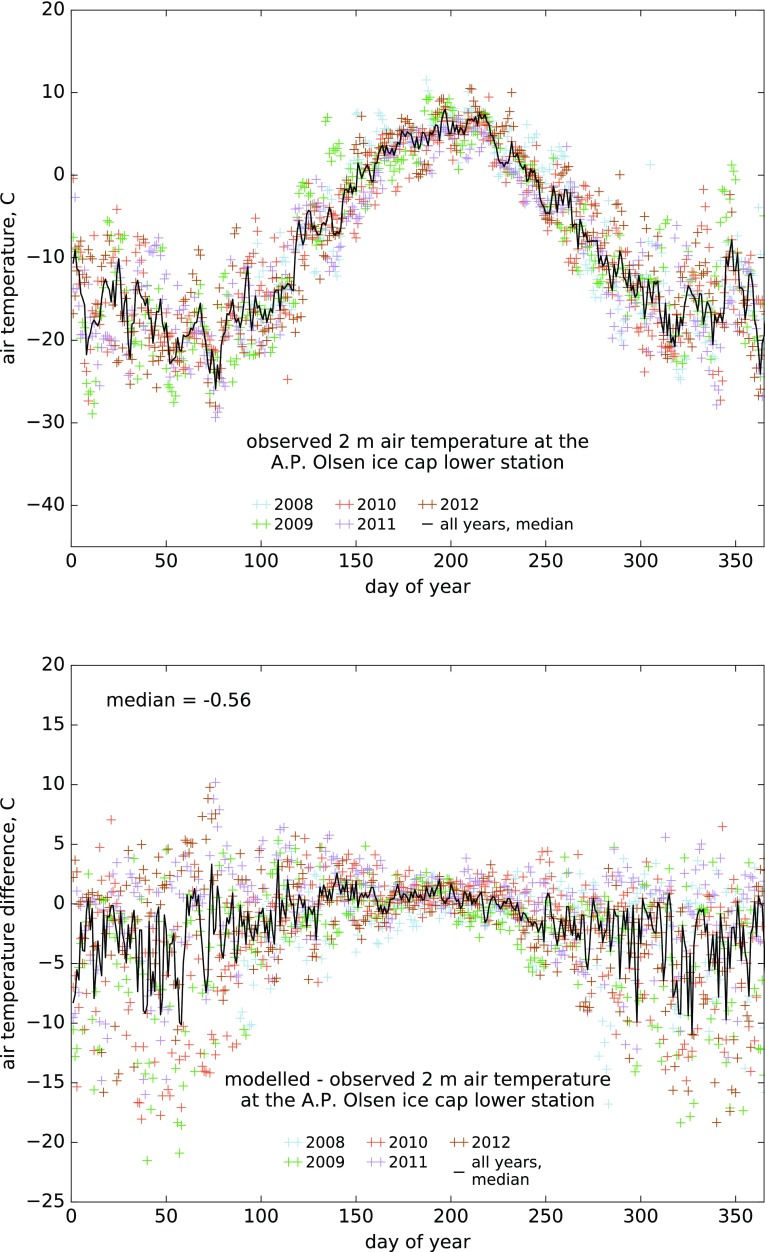
Observed daily average 2-m air temperature at the A.P. Olsen ice cap lower AWS, 2008–2012 (**a**) and difference of modelled versus observed temperature (**b**)

Downscaled HIRHAM5 modelled 2-m air temperature reproduces observations well, particularly over glacier surfaces where the median daily bias over the 2008–2012 period is −0.56 °C (Fig. 2). Without correcting for elevation bias, the median daily bias over the same period would be −2.01 °C and the buffering effect of the melting surface would be less pronounced (Fig. S3). At the climate mast close to Zackenberg Research Station, the median downscaled model bias is −1.54 °C (1996–2012), with a larger variability of daily average 2 m temperature around the median of the period (Fig. S4). At both sites, the variability of daily average 2 m temperature and model bias are larger during the cold season, when similar or lower temperatures than at the glacier AWS (660 m a.s.l.) are commonly observed at the lower lying climate mast close to sea level.

**Fig. 3:**
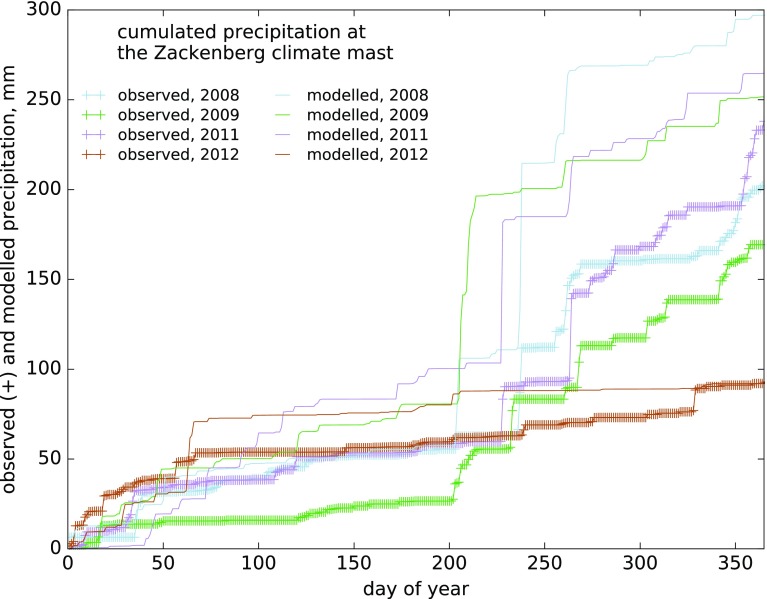
Comparison of observed and modelled cumulated daily precipitation curves at the site of the climate mast close to Zackenberg Research Station, 2008–2012 (2010 is not shown due to a large data gap)

At the site of the Zackenberg climate mast, the HIRHAM5 model appears to overestimate annual precipitation totals (Fig. 3), primarily by producing a few much larger precipitation events than observed during July and August, which can account for most of the annual overestimation (2008, 2009 and 2011). Except for these extreme events, the model also seems to produce more precipitation in the first half of the year than observed, even in years with annual totals close to observed (2012).

**Fig. 4:**
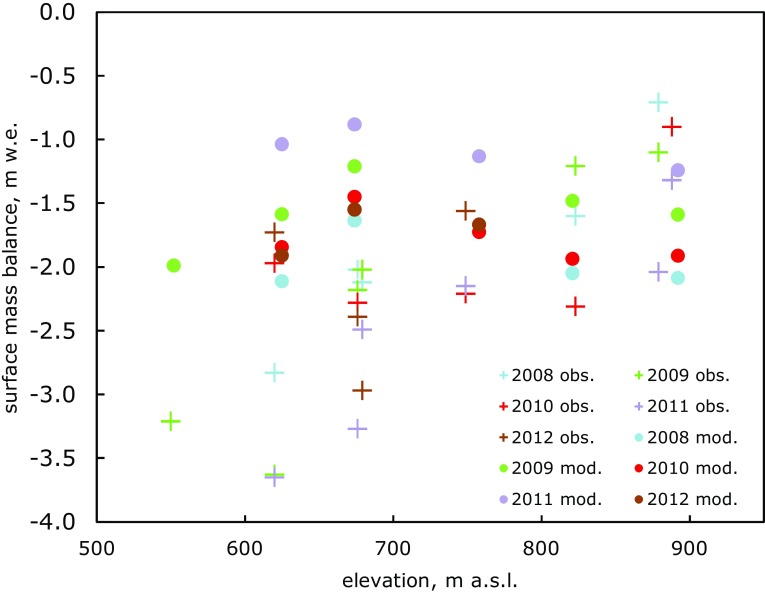
Annual observed and modelled surface mass balance for A.P. Olsen ice cap ablation area stakes 1 (550 m a.s.l.) to 9 (888 m) between 2008 and 2012

Glacier ablation estimated from the model is consistently biased towards less negative annual mass balance and a less steep mass balance gradient below 700 m a.s.l. than observed (Fig. 4) in agreement with Langen et al. (accepted). Above 700 m, the modelled mass balance is closer to observed, but the mass balance gradient is not well defined.

**Fig. 5:**
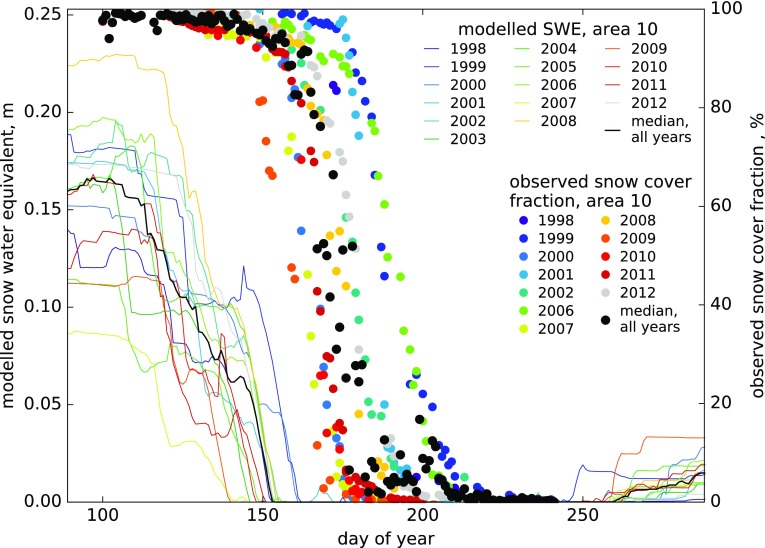
Observed snow-covered area fraction and modelled SWE, 1998–2012: seasonal depletion curves for ‘area 10’ (similar curves for a higher and a lower average snow areas are shown in Fig. S5)

**Fig. 6:**
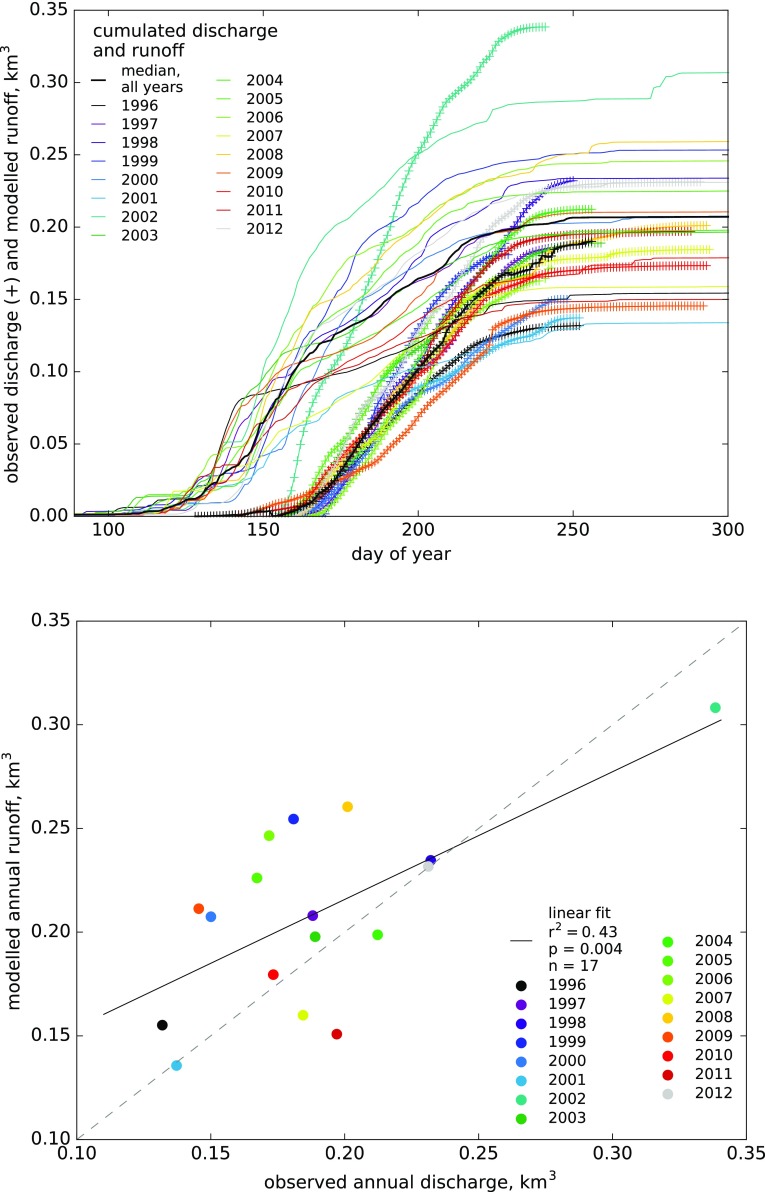
Observed river discharge and modelled runoff, 1996–2012: cumulative daily curves (**a**) and comparison of year totals, with the statistically significant linear fit marked by the* solid black line* (**b**)

Comparing SWE against observed snow-covered area fraction in ‘area 10’ (Fig. 5), ‘area 11’ and ‘central area’ (Fig. S5) shows that the modelled seasonal snow cover always starts depleting and disappears more than a month earlier than observed. This happens consistently in all years with available observations (1998–2012). This early modelled start of the melt season is also visible in the comparison of 1996–2012 daily model runoff versus observed Zackenberg River discharge (Fig. 6a). The modelled cumulative runoff tends to show higher discharge (steeper cumulative curve) than observed in the first half of the melt season, followed by lower discharge than observed during the rest of the season (Fig. 6a). The correlation between annual modelled runoff and observed river discharge is significant (Pearson product moment correlation, *n* = 17, *P* < 0.005, Fig. 6b), but the runoff model only predicts 43% of the variance in observed annual total discharge. For 2006, our model estimates a total annual runoff of 1.3 km^3^ which agrees with the 0.9–1.4 km^3^ range estimated by Bendtsen et al. (2014).

**Fig. 7:**
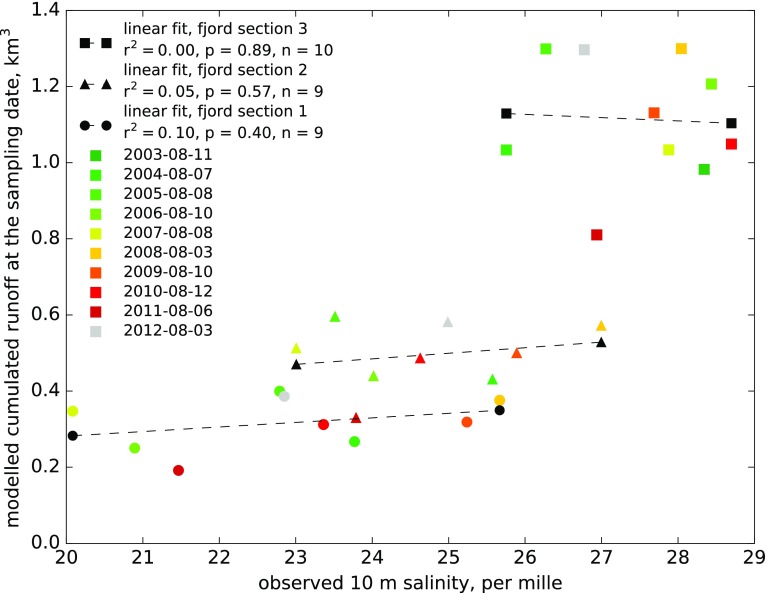
Lack of significant correlation between modelled runoff and observed 0–10 m salinity for the 3 sections of Tyrolerfjord–Young Sound corresponding to the three terrestrial catchments modelled

**Fig. 8:**
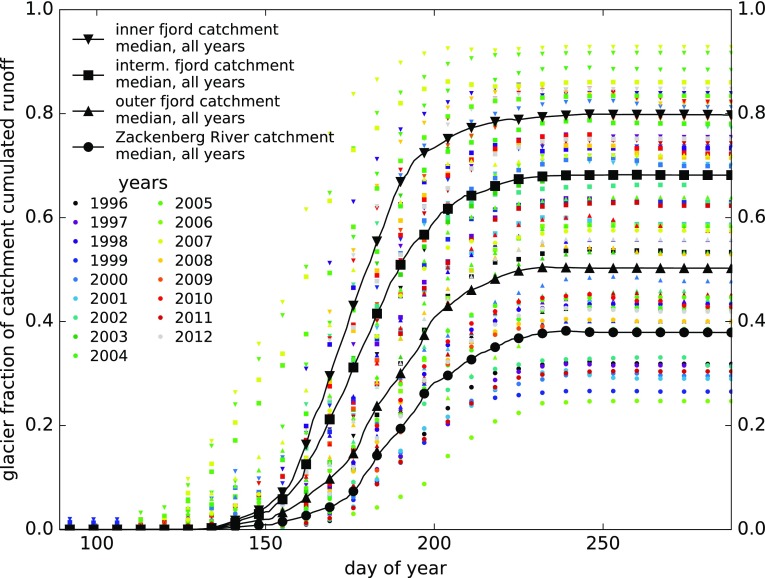
Model estimate of the fraction of terrestrial runoff originating from glaciers

The interannual variation in average salinity of the surface layer (0–10 m) for each of the four fjord sections is given in Table 1. Surface salinity does not display a clear long-term trend across any of the four sections. But, lower and more variable surface salinity is found in sections 1 and 2 which largely vary in synchrony. Minimum salinities are found in 2007, 2011 and 2015. In section 3, salinity is higher, less variable and shows a different interannual pattern than sections 1 and 2 indicating that other factors are important for surface salinity in section 3 compared to sections 1 and 2. Runoff from land creates a low salinity surface layer throughout the Tyrolerfjord–Young Sound fjord system. Most of the freshwater from land is found in a 6–8 m thick surface layer due to its lower density (Fig. S6). Here salinity is lower than the water mass below and temperatures are higher—often above 10 °C at the surface (data not shown). Salinity in the inner part of Tyrolerfjord (section 1) shows a strong influence of glacial melt water in the Tyroler Elv river and other smaller rivers contributing with glacial melt water from the Greenland ice sheet and local ice caps (Fig. 8). This section typically shows the lowest salinity in the surface water (Figs. 7, 8 and Fig. S7), which gradually increases towards the sea (section 4). In sections 3 and 4, a larger fraction of freshwater originates from land runoff north of Young Sound combined with melting sea ice. Correlation analysis between the time series in salinity from each of the four sections showed that only sections 1 and 2 were significantly correlated (*n* = 12, *R* ^2^ = 0.73, *P* < 0.001) and thus displayed similar interannual variation. This indicates that in sections 1 and 2, surface salinity is showing similar year-to-year variation and thus likely influenced by the same overall processes. In contrast, sections 3 and 4 are increasingly influenced by the ocean and contribution of freshwater from melting sea ice and runoff north of our study area. We thus combined sections 1 and 2 and calculated the average salinity each year for this inner part of the fjord corresponding to the intermediate catchment area, where we would expect runoff to exert the strongest control on surface salinity. Based on the overlapping time series in summer salinity and modelled runoff from 2004 to 2012, we would expect a negative relationship between runoff (we used accumulated runoff until the date of the fjord sampling took place) and summer salinity. However, the linear regression shows a positive relationship although it is not statistically significant (*P* = 0.22, *n* = 9). Significant linear regressions between runoff and fjord salinity were not found for any of the other sections. For the combined sections 1 and 2, we also calculated average salinity for the 0–5 m surface layer depth to test if the lack of relationship to runoff was sensitive to the thickness of the surface layer. However, the time series based on the 0–5 m showed similar overall patterns as the time series based on the 0–10 m surface layer and the two were closely correlated (*n* = 12, *R* ^2^ = 0.87, *P* < 0.001). Using the 0–5 m data series did not provide a significant relationship to the modelled runoff and neither did using the estimated runoff for the 21 days prior to the fjord sampling.

**Table 1:** Average salinity at 1–10 m depth in the four fjord sections (refer to Fig. 1 for their position)

Year	2003	2004	2005	2006	2007	2008	2009	2010	2011	2012	2013	2014	2015
Date of sampling	Aug-11	Aug-07	Aug-08	Aug-10	Aug-08	Aug-03	Aug-10	Aug-12	Aug-06	Aug-03	Aug-12	Aug-03	Aug-16
Section 1	n/a	23.77	22.79	20.89	20.08	25.67	25.24	23.36	21.47	22.85	25.86	25.28	21.59
Section 2	n/a	25.57	23.51	24.02	23.01	27.00	25.89	24.63	23.79	24.99	25.24	25.69	22.46
Section 3	28.34	25.75	26.27	28.44	27.88	28.04	27.69	28.70	26.94	26.77	26.99	27.93	27.32
Section 4	29.37	29.44	26.75	29.40	27.51	29.57	28.93	30.20	29.74	29.77	28.35	29.24	28.89

The modelled fraction of terrestrial runoff originating from glaciers grows during the ablation season in all modelled catchments (Fig. 8), with glaciers in the inner fjord catchment delivering as much as 80% of the terrestrial freshwater input of fjord section 1. The terrestrial runoff from the intermediate and outer catchments is composed by 70% and 50% of glacier runoff, respectively, and this figure is slightly less than 40% for the Zackenberg River catchment, which includes a comparatively smaller glacierized area (Fig. 8). This result for Zackenberg River is substantially lower than the 74% average for 1998–2004 modelled by Mernild et al. (2008).

## Discussion

In the investigated region, the performance of downscaled HIRHAM5 compared to in situ observations is mixed, with good results for 2 m air temperature, overestimation especially of large precipitation events, and anticipated start of ablation and runoff compared to field evidences. The model does not include routing of surface water through the drainage network, which for all of our investigated catchments will result in faster modelled runoff to the sea than in reality. However, this would not explain our finding of a similar (month-scale) difference in observed versus modelled disappearance of the snow cover over areas smaller than a single HIRHAM5 cell. The HIRHAM5 products used in this study include a subsurface scheme for meltwater retention which reduces and delays the runoff from glaciers. However, this scheme is not implemented over land, which certainly results in the modelled snowpack outside glaciers disappearing too rapidly. The modelled runoff from the Zackenberg River catchment being higher than observed river discharge in the early season and lower in the late season (Fig. 6) is consistent with this interpretation, as is the later start of strong modelled runoff (Fig. S7) to fjord sections 1 and 2, which receive most of their annual freshwater input from glaciers (Fig. 8), compared to section 3. A timing error of modelled terrestrial runoff in the order of a month can explain the surprising lack of a significant negative correlation of observed fjord surface salinity with modelled terrestrial runoff, because freshwater at the surface is estimated to have a residence time of only 10–30 days (Bendtsen et al. 2014).

A different potential explanation could be that our measurement of salinity is somehow biased and does not capture the actual interannual variation in freshwater content. However, our time series of surface salinity is not very sensitive to changing to depth used to calculate average salinity (upper 5 or 10 m) and the two inner sections of the fjord also displayed very similar interannual variation, suggesting that this time series in fact is a robust estimate of the freshwater in the surface layer in this part of the fjord.

If the model is biased towards too early depletion of the snow cover outside glaciers, the modelled fraction of terrestrial runoff originating from glaciers may evolve during the season following a different curve than modelled but reaching the same end of year values (Fig. 8). It is important in this respect to also consider the model performance in reproducing observed glacier mass balance, because we found that the model may be underestimating glacier mass loss at least in the ablation area. This assessment is complicated by one of the two HIRHAM5 grid cells covering all of our ablation stakes on A.P. Olsen ice cap being modelled as land rather than ice. The other cell is modelled at an elevation above the equilibrium line, while the stakes it covers are in fact at lower elevation in the upper ablation zone. This would also complicate de-biasing modelled ablation based on stake observations. De-biasing to match observed Zackenberg River discharge is further hampered by the discussed timing mismatch of snowpack depletion and the large glacier-free part of the catchment between A.P. Olsen and the hydrometric station. However, neither the exact timing of seasonal snowpack depletion nor a possibly underestimated glacier ice ablation can alter the overall picture of terrestrial runoff being dominated by glaciers for fjord sections 1 and 2 (‘inner’ and ‘intermediate’ catchments). Similarly, glacier runoff would still be a large component in section 3 and in Zackenberg River, and the relative importance of glacier runoff would still reach a maximum at the end of the melt season (Fig. 8).

## Conclusions

Runoff is a central driver for marine biogeochemical processes in Greenland fjords. At this point, much of our understanding of terrestrial runoff and how melt water impacts marine ecosystems in Greenland fjords originates from the two fjord systems where the GEM monitoring programmes have provided logistics, funding and data supplemented by focused research programmes. Established RCMs known to perform well over large ice masses like the Greenland ice sheet can be less accurate over the comparatively narrow transition zone between the ocean and the ice sheet, especially in regions characterized by steep and complex topography like the one discharging freshwater into Tyrolerfjord and Young Sound. The unique availability of extensive terrestrial and marine in situ observations made it possible to investigate the performance of HIRHAM5 over this region through a simple downscaling scheme. This provided an estimate of the glacial runoff signal in the terrestrial freshwater input to the sea, and its seasonal evolution, that are robust against the identified weaknesses of the available information.

The HIRHAM5 model currently represents state of the art in terms of high-resolution RCM in Greenland, as it is one of the very few models run at a spatial resolution of 5.5 km. However, the complex topography in the Zackenberg region likely requires even higher spatial resolution and sophisticated non-hydrostatic model dynamics in order to accurately model complex land–ice–ocean interactions on short timescales (Mottram et al., unpubl.) Ongoing development of HIRHAM5 is expected to improve the modelling of runoff from glaciers and from land, and allow detecting the terrestrial freshwater signal in the surface fjord salinity. Providing a first estimate of the runoff in other regions of Greenland will be the next step in trying to test and upscale to larger regions of Greenland the understanding gathered at the GEM sites in Zackenberg/Young Sound and in Godthaabs fjord.

## Electronic supplementary material

Below is the link to the electronic supplementary material.

Supplementary material 1 (PDF 3395 kb)
