# Supplementary material for: Towards quantifying the glacial runoff signal in the freshwater input to Tyrolerfjord–Young Sound, NE Greenland
Source: Ambio. 2017 Jan 23;46(Suppl 1):146–59. doi: 10.1007/s13280-016-0876-4 (PMC5258666; doi:10.1007/s13280-016-0876-4)
Supplement: Supplementary file 1 — Supplementary material 1 (PDF 3395 kb) [file 13280_2016_876_MOESM1_ESM.pdf]

Ambio

Electronic Supplementary Material
This supplementary material has not been peer reviewed.

Title: Towards quantifying the glacial runoff signal in the freshwater input to Tyrolerfjord-Young Sound, NE Greenland

Autors: Michele Citterio, Mikael K. Sejr, Peter Lang Langen, Ruth H. Mottram, Jakob Abermann, Signe Hillerup Larsen, Kirstine Skov, Magnus Lund.

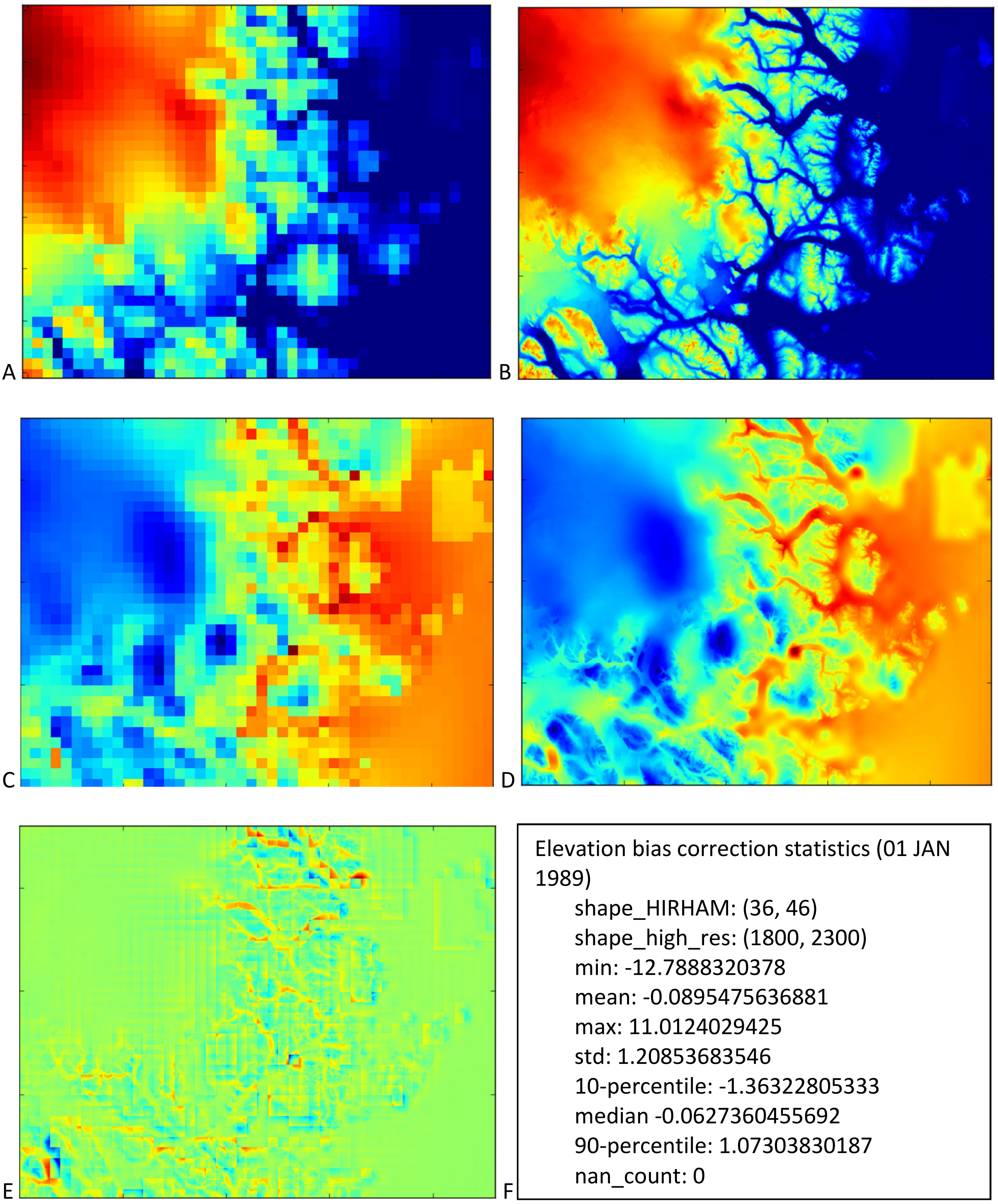

Fig. S1: Visualization of downscaling and elevation bias correction of 2m air temperature from the coarse HIRHAM5 grid to the high resolution grid used in this work. Surface elevation in HIRHAM5 is shown in A, and resampled from the GIMP DEM in B. The original HIRHAM5 2m air temperature for the day 1/1/1989 is shown in C, and the downscaled and elevation bias-corrected version produced by our model is shown in D. The calculated correction values applied to C when producing D are shown in panel E, with statistics showing this correction can exceed 10 C in either direction within some HIRHAM cells without introducing any regional bias (mean and median correction ≈ 0 C).

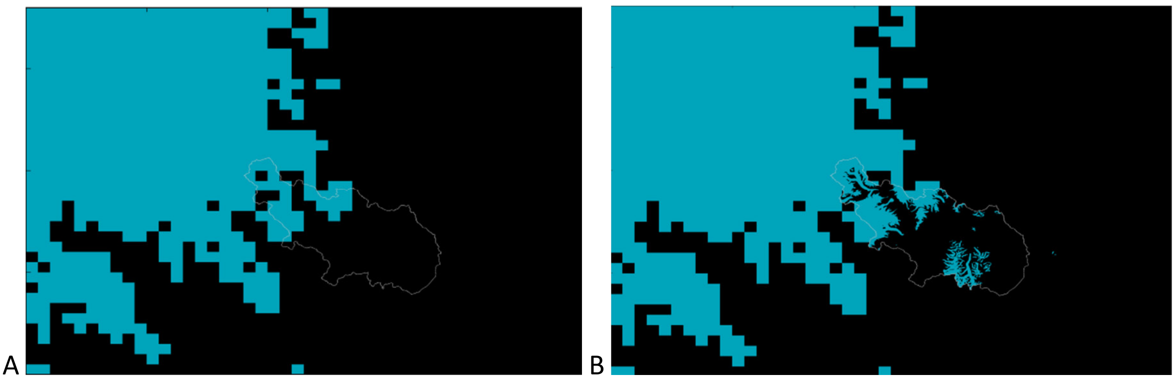

A B

Fig. S2: Visualization of surface cover type correction while downscaling glacier mass balance from the coarse HIRHAM5 grid to the high resolution grid used in this work. The glacier mask in HIRHAM5 is shown in A, and refined to match the PROMICE glacier mask of Citterio & Ahlstrøm, 2013 in B. The thin white line outlines the Tyrolerfjord catchment (see Fig 1). Note the large glaciers on Clavering Island and the A.P. Olsen outlet glacier monitored by GlacioBasis are now included.

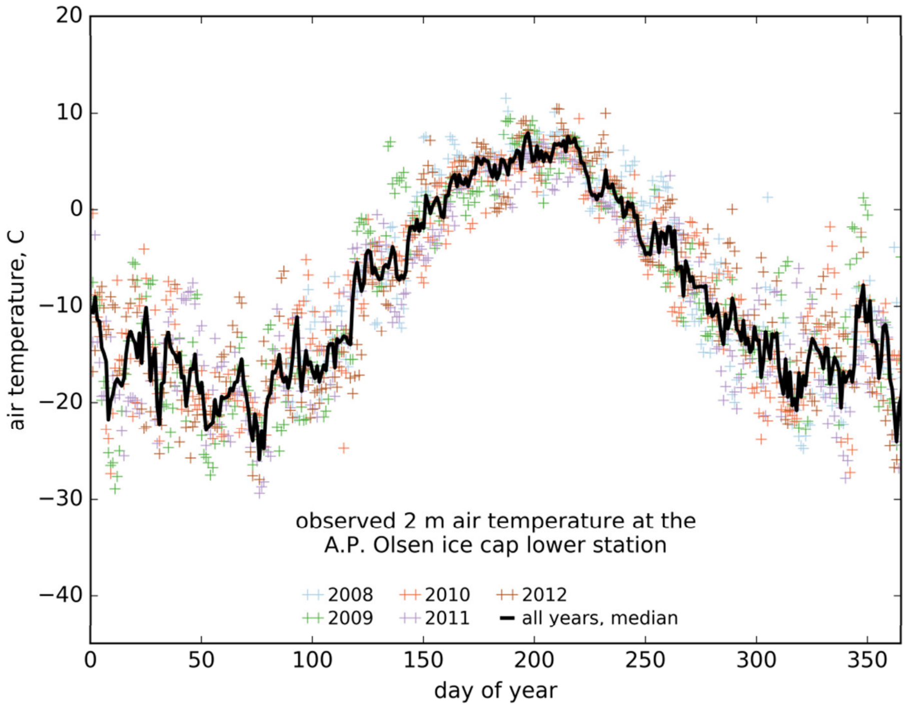

A

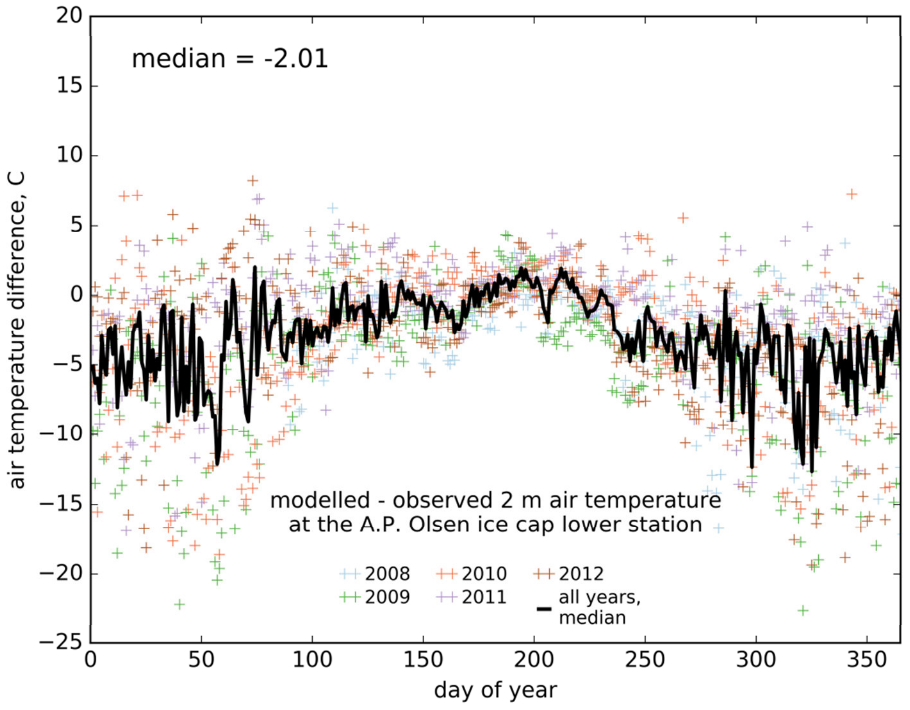

B

Fig. S3 – Observed 2 m air temperature at the site of the lower A.P. Olsen AWS (A) and error of the modelled temperatures (B). This figures is analogous to Fig. 2 but without correcting for elevation bias.

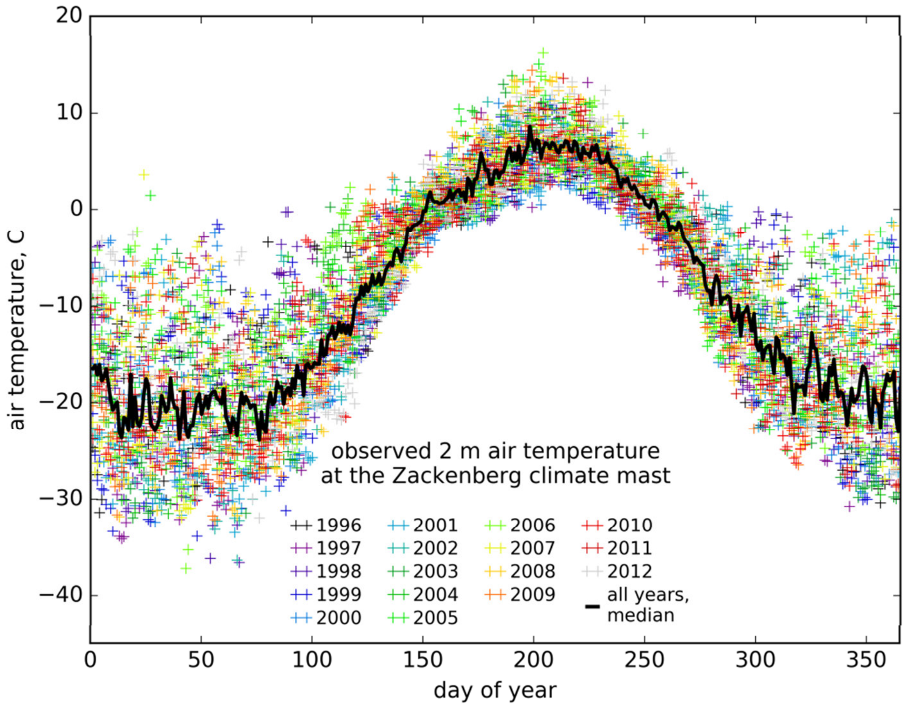

A

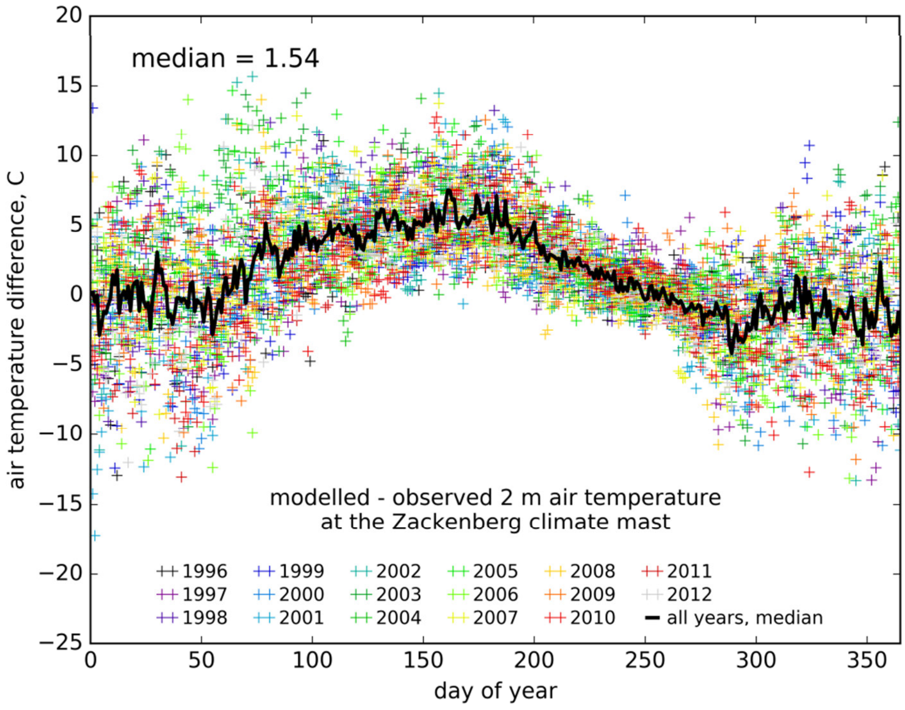

B

Fig. S4 – Observed 2 m air temperature at the site of the Zackenberg climate masts (A) and error of the modelled temperatures (B).

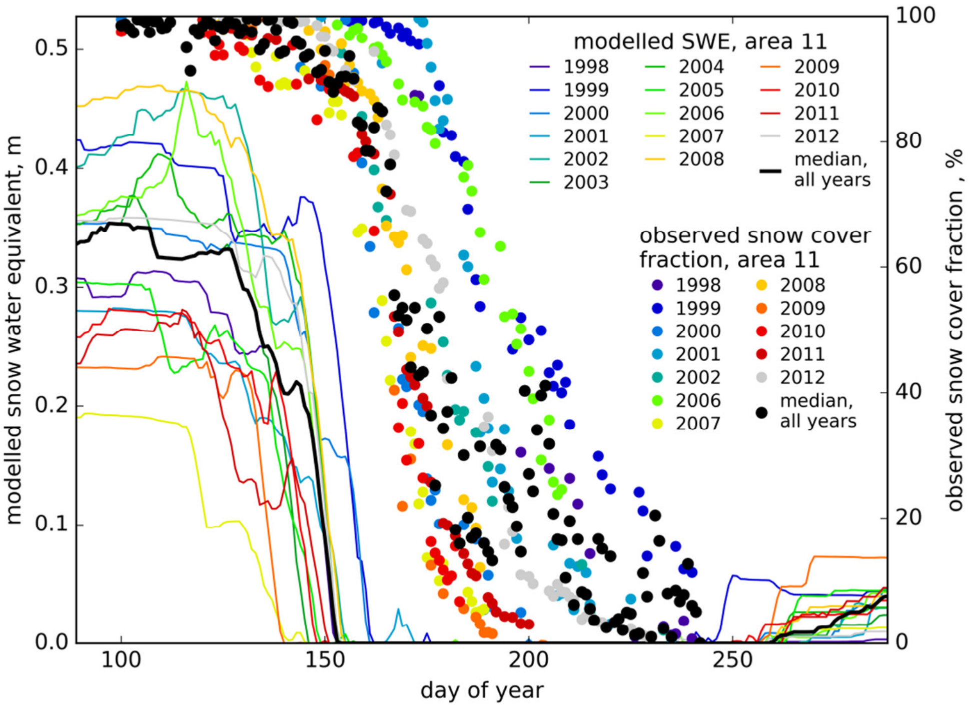

A

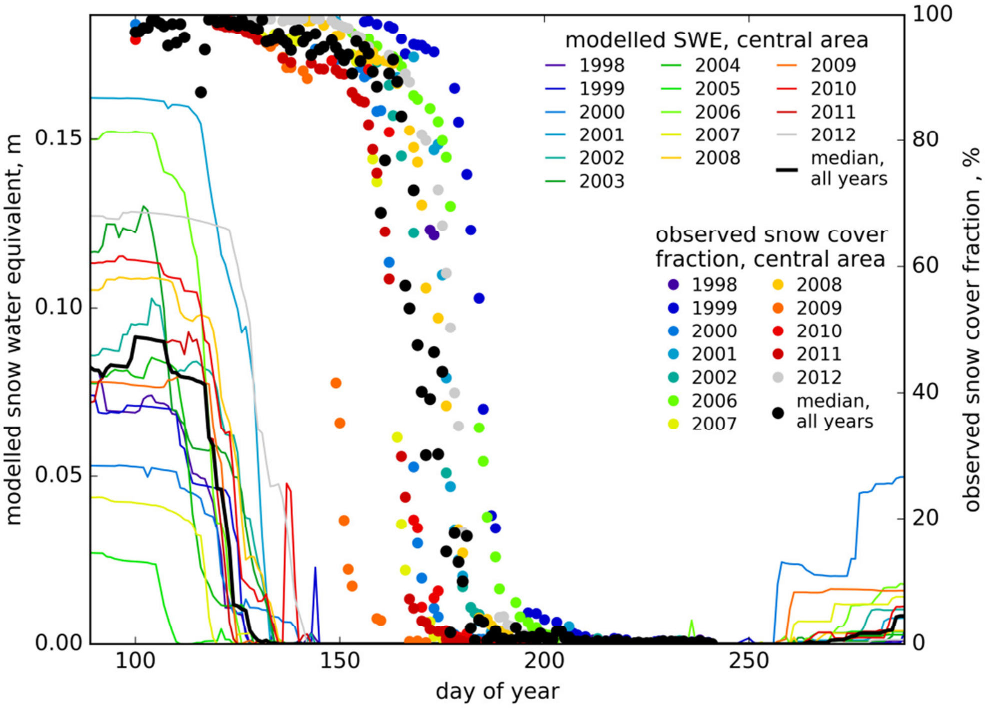

B

Fig. S5 - Observed snow covered area fraction and modelled SWE, 1998-2012: seasonal depletion curves for ‘area 11’ (A) and ‘central area’ (B), analogous to Fig. 5 (‘area 10’).

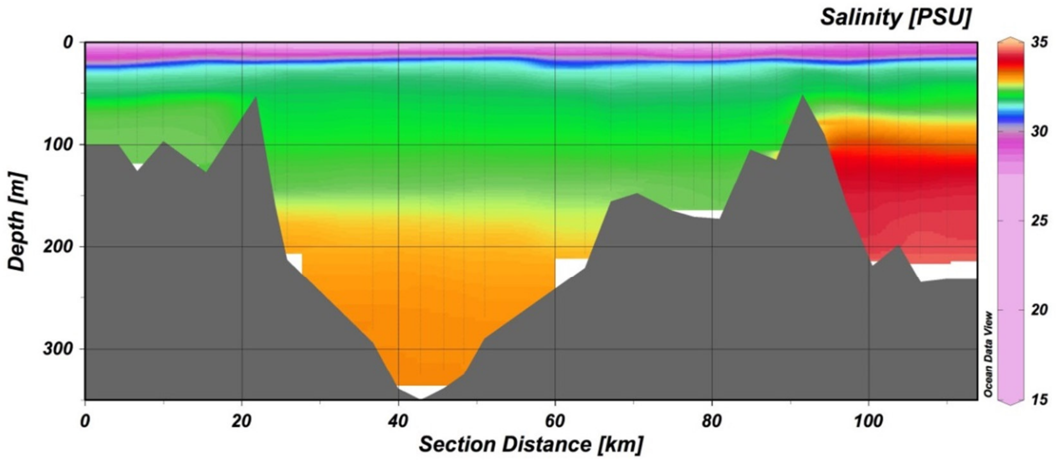

Fig S6: Distribution of salinity in the Tyrolerfjord-Young Sound fjord system in August 2011

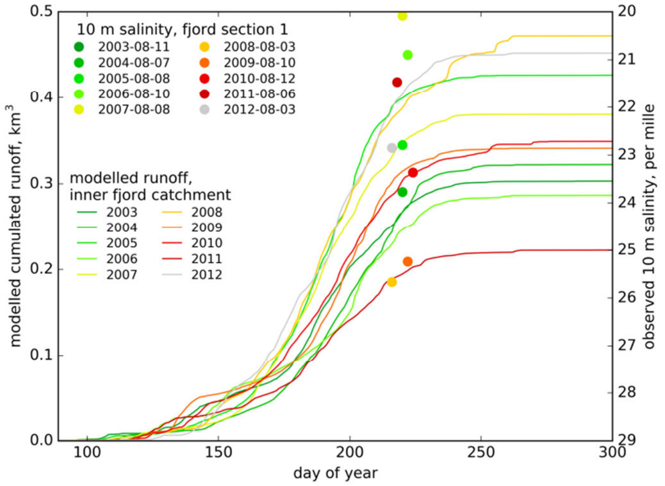

A

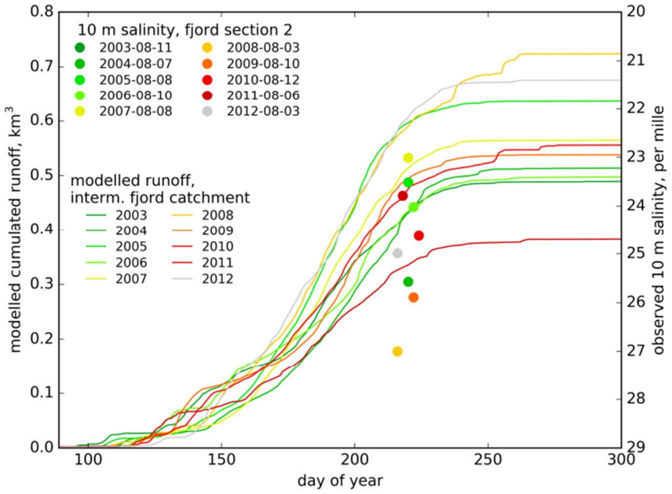

B

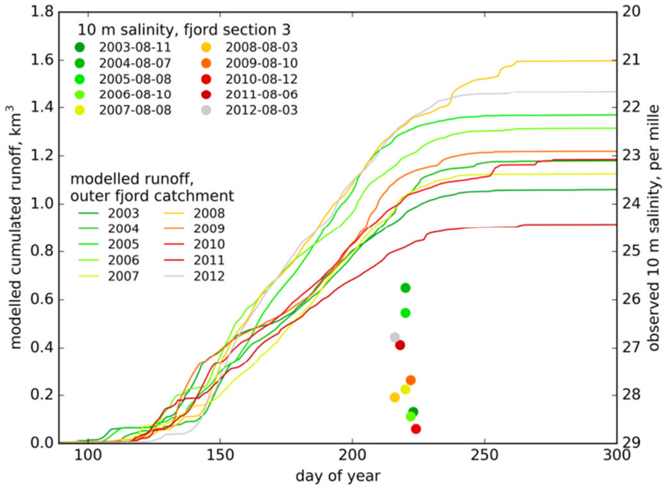

C

Fig. S7 average salinity of the surface layer (0-10 m) at the date of sampling are marked by dots for fjord section 1 to 3 (panel A to C, respectively). The modelled cumulated daily runoff curves are also shown for the corresponding terrestrial catchments.

REFERENCES

Citterio, Michele, and Andreas P. Ahlstrøm. ‘Brief communication “The Aerophotogrammetric Map of Greenland Ice Masses”’. The Cryosphere 7, no. 2 (11 March 2013): 445–49. doi:10.5194/tc-7-445-2013.
